# Perfrin, a novel bacteriocin associated with *netB* positive *Clostridium perfringens* strains from broilers with necrotic enteritis

**DOI:** 10.1186/1297-9716-45-40

**Published:** 2014-04-05

**Authors:** Leen Timbermont, Lina De Smet, Filip Van Nieuwerburgh, Valeria R Parreira, Gonzalez Van Driessche, Freddy Haesebrouck, Richard Ducatelle, John Prescott, Dieter Deforce, Bart Devreese, Filip Van Immerseel

**Affiliations:** 1 Department of Pathology, Bacteriology and Avian Diseases, Research Group Veterinary Public Health and Zoonoses, Faculty of Veterinary Medicine, Ghent University, Salisburylaan 133, B-9820 Merelbeke, Belgium; 2 Department of Biochemistry and Microbiology, Faculty of Sciences, Ghent University, K. L. Ledeganckstraat 35, 9000 Ghent, Belgium; 3 Lab for Pharmaceutical Biotechnology, Faculty of Pharmaceutical Sciences, Ghent University, Harelbekestraat 72, 9000 Ghent, Belgium; 4 Current address: Department of Physiology, Faculty of Sciences, Ghent University, K. L. Ledeganckstraat 35, 9000 Ghent, Belgium; 5 Department of Pathobiology, University of Guelph, Guelph, N1G 2 W1, Ontario, Canada

## Abstract

Necrotic enteritis in broiler chickens is associated with *netB* positive *Clostridium perfringens* type A strains. It is known that *C. perfringens* strains isolated from outbreaks of necrotic enteritis are more capable of secreting factors inhibiting growth of other *C. perfringens* strains than strains isolated from the gut of healthy chickens. This characteristic could lead to extensive and selective presence of a strain that contains the genetic make-up enabling to secrete toxins that cause gut lesions. This report describes the discovery, purification, characterization and recombinant expression of a novel bacteriocin, referred to as perfrin, produced by a necrotic enteritis-associated *netB*-positive *C. perfringens* strain. Perfrin is a 11.5 kDa C-terminal fragment of a 22.9 kDa protein and showed no sequence homology to any currently known bacteriocin. The 11.5 kDa fragment can be cloned into *Escherichia coli*, and expression yielded an active peptide. PCR detection of the gene showed its presence in 10 *netB*-positive *C. perfringens* strains of broiler origin, and not in other *C. perfringens* strains tested (isolated from broilers, cattle, sheep, pigs, and humans). Perfrin and NetB are not located on the same genetic element since NetB is plasmid-encoded and perfrin is not. The bacteriocin has bactericidal activity over a wide pH-range but is thermolabile and sensitive to proteolytic digestion (trypsin, proteinase K). *C. perfringens* bacteriocins, such as perfrin, can be considered as an additional factor involved in the pathogenesis of necrotic enteritis in broilers.

## Introduction

Enteric diseases are an important concern to the poultry industry because of production losses, increased mortality, reduced welfare of birds and increased risk of contamination of poultry products for human consumption. Necrotic enteritis is a widespread disease in broilers imposing a significant economic burden on the poultry industry worldwide. The total global economic loss as a consequence of necrotic enteritis outbreaks in broiler farms is estimated to be over 2 billion dollars annually [1,2].

*Clostridium perfringens* type A is widely prevalent in the environment and in the intestinal tract of animals and humans. Necrotic enteritis in poultry is associated with a specific subpopulation of *C. perfringens* type A, i.e. strains carrying the NetB toxin [3,4]. As a consequence, strains isolated from outbreaks of necrotic enteritis are able to induce lesions in an experimental model using predisposing factors, in contrast to strains isolated from the gut of healthy broilers [5,6]. *NetB* resides in a large plasmid encoded locus [7].

In *C. perfringens* isolates from healthy birds, a high degree of genetic diversity can be found, even between isolates within the gut of a single animal. In contrast, in a flock suffering from a clinical outbreak, different isolates are generally of the same pulsed-field gel electrophoresis (PFGE) type, regardless of which animal in the flock or which part of the intestine the strain is isolated from [8,9]. Recent data show that *C. perfringens* is capable of secreting factors inhibiting growth of other *C. perfringens* strains. The intra-species inhibitory phenotype was shown to be more prevalent in outbreak strains compared to strains isolated from the gut of healthy broilers [10,11]. This characteristic could allow a single strain to outcompete other *C. perfringens* strains in the gut, and if it contains the genetic make-up enabling to secrete toxins, it could consequently cause gut lesions.

Many *C. perfringens* strains are able to produce antibacterial proteins, called bacteriocins [12]. Bacteriocins are proteinaceous toxic compounds produced by bacteria that generally inhibit the growth of closely related strains [13], and are thus potential candidates involved in the intra-species inhibitory phenotype of *C. perfringens*. The nature of the inhibitory components that cause intra-species growth-inhibition in broiler outbreak strains of *C. perfringens* was hitherto unknown. In the present study, we purified and characterized a novel antimicrobial peptide from a *C. perfringens* outbreak strain with intra-species inhibitory activity and showed it to be located on the chromosome and specifically present in a selection of *netB* positive *C. perfringens* strains from broilers.

## Materials and methods

### Strains

Fifty *C. perfringens* type A strains isolated from broiler chickens belonging to different genotypes, as analyzed by pulsed-field gel electrophoresis (PFGE), were included. Thirty-five strains were isolated from broiler chickens in Belgium: 26 strains from clinically healthy broiler chickens and 9 strains from broilers suffering from necrotic enteritis [9]. Fifteen Danish *C. perfringens* isolates from necrotic enteritis cases were kindly provided by Dr L. Bjerrum [8]. *C. perfringens* strain 56 was isolated from the intestine of a broiler chicken with severe necrotic gut lesions. It was selected because it is a virulent strain that inhibits the growth of 41 of the 50 strains used [5,11]. Strain 6 was isolated from the normal gut microbiota of a healthy broiler chicken and was used as indicator strain because it is not able to inhibit other *C. perfringens* strains and its growth is inhibited by strain 56 [11].

Forty-five randomly chosen *C. perfringens* strains of different origin were used in the *perfrin* PCR: ATCC3624; NCTC3110; NCTC3180; NCTC8503; NCIB 10748; 13 strains isolated from cattle; 11 from pigs; 10 from turkeys, three from sheep and three from humans.

*C. perfringens* strain CP4, a strain isolated from a broiler suffering from necrotic enteritis, was used in Southern Blotting experiments [14].

### Agar spot test

Bacteria to be tested were grown overnight anaerobically in Brain Heart Infusion (BHI, Oxoid, Basingstoke, UK) broth. Lawns of bacteria were prepared by diluting the overnight cultures in phosphate buffered saline (PBS) to a density of McFarland 1, and 100 μL of these suspensions were spread with a sterile swab on the surface of BHI agar plates. Drops of 20 μL of the fractions with potential antimicrobial activity (see below) were spotted on these lawns or single colonies of *C. perfringens* strains were transferred with a sterile toothpick to the agar plates. Antimicrobial activity is shown by the absence of growth of the bacterial lawn around the colony or on the location of the spot.

### Antimicrobial protein purification and identification

*C. perfringens* strain 56 was grown anaerobically in Tryptic Soy Broth (TSB, Oxoid) for 24 h at 42 °C (800 mL). Culture supernatant was obtained by centrifugation at 17 000 *g* for 15 min at 4 °C. The supernatant was filter sterilized (0.22 μm) and supernatant proteins were precipitated by overnight incubation in 50% (w/v) (NH_4_)_2_SO_4_ (Sigma Aldrich, St. Louis, MO, USA) at 4 °C followed by centrifugation at 17 000 *g* for 2 h at 4 °C. The precipitate was resuspended in 5 mL of PBS and dialyzed against 10 mM Tris–HCl buffer, pH 8.5 (5 L) for 24 h at 4 °C with 3 buffer changes. This concentrated supernatant was tested for antimicrobial activity against *C. perfringens* strain 6 using the agar spot test. Antimicrobial concentrated supernatant was loaded onto a SP-Sepharose cation exchange column (GE Healthcare, Little Chalfont, Buckinghamshire, UK) in 10 mM NaOAc buffer, pH 4. Proteins were eluted using stepwise increasing concentrations of NaCl (up to 1 M). To determine the antimicrobial activity of the fractions, two-fold dilutions of the fractions were spotted on a lawn of strain 6 in the agar spot test (see above). The most active fractions in the antimicrobial assay were pooled and loaded onto a butyl Sepharose hydrophobic interaction column (GE Healthcare) in 50 mM sodium phosphate buffer with 1 M (NH_4_)_2_SO_4_, pH 7.4. Proteins were stepwise eluted from the column with decreasing concentrations of (NH_4_)_2_SO_4_. The activity of the fractions was again determined and the most active fraction was concentrated and analyzed by SDS-PAGE. Precision Plus Protein Standard, All Blue (Bio-Rad Laboratories, Hercules, CA, USA) was used as protein marker. The protein band was cut from the gel and subjected to in-gel protein digestion with trypsin [15] followed by mass spectrometric characterization. After mixing 1 μL of the digestion mixture with 10 μL α-cyano 4-hydroxycinnamic acid (5 mg/mL), 1 μL was spotted onto the target plate and analyzed with the 4800 plus MALDI TOF/TOF Analyzer (Applied Biosystems, Foster City, CA, USA). Mass spectral data were searched against different protein databases using an in-house MASCOT server (Matrixscience, London, UK). A NCBI BLAST-search was done with the amino acid sequences revealed by manual interpretation of the MS/MS spectra [16]. The protein fragment sequences were also compared with the genome sequence of strain 56. Prediction of transmembrane helices was done with TransMembrane prediction using Hidden Markov Model (TMHMM) [17]. Prediction of signal peptides was done with Signal-BLAST [18] and Sigcleave [19]. ExPASy was used [20] to determine the theoretical isoelectric point and molecular weight.

### Genome sequencing of *C. perfringens* strain 56

5 μg of genomic DNA was extracted and purified using Easy DNA kit (K1801, Invitrogen, Merelbeke, Belgium). Roche GS-FLX titanium libraries were generated, using 5 μg of the purified DNA sample. The DNA was fragmented by nebulisation, followed by a double Solid Phase Reversible Immobilization (SPRI) bead capture size selection with Ampure beads (Agencourt Bioscience, Beverly Massachusetts, USA) to generate DNA fragments of 400–1.500 bp in length. Selected fragments were end-repaired and ligated to 454 sequencing adapters. A single stranded library was then generated according to the Roche GS FLX Titanium General Library Preparation Method Manual (version October 2008). This single stranded library was used to perform an emulsion PCR according to the Roche GS FLX titanium emPCR Method Manual (version October 2008).

The resulting bead library was sequenced on a Roche GS-FLX system following the GS FLX Titanium Sequencing Method Manual (version October 2008). A 70 × 75 mm picotiter plate was divided in 2 lanes using a rubber gasket. One of the two lanes was loaded with 2 million DNA library beads.

The Mimicking Intelligent Read Assembly package MIRA (version 2.9.58) [21] was used to perform a *de novo* genome assembly. MIRA used 550.692 of the 556.703 generated sequences to assemble 59 relevant contigs, ranging from 226.570 to 2.627 bp. The total consensus sequence was 3.633.567 bp, with an average sequencing coverage of 57X. The contigs were mapped against the reference genome “*Clostridium perfringens* ATCC 13124 complete genome” (gi|110798562|ref|NC_008261.1) using Projector 2 [22]. This mapping allowed for a manual scaffolding of overlapping contigs, resulting in 29 scaffolds ranging from 481.823 to 4.838 bp. Mapping of these scaffolds against “*Clostridium perfringens* ATCC 13124 complete genome” using Projector 2 showed an almost completely closed circular genome, with only 28 small gaps (< 20.035 bp) and a total consensus sequence of 3.290.797 bp.

All relevant scaffolds were submitted to the Rapid Annotations using Subsystems Technology server (RAST) [23] for a fully automated annotation of the sequences.

### Amino acid sequence accession number

The amino acid sequence data reported in this manuscript are available from GenBank under accession number HQ666823.

### Detection of *perfrin* in *C. perfringens* strains

The presence of the *perfrin* gene in the 50 poultry *C. perfringens* strains and 45 strains from different origin was investigated by PCR. One colony from each strain was suspended in 20 μL lysis buffer (0.25% SDS, 0.05 N NaOH), heated at 95 °C for 5 min, diluted by adding 180 μL of pure water and then centrifuged at 10 000 *g* for 5 min. The supernatants were collected and used as the template for PCR. The reactions were performed in a total volume of 10 μL, containing 1 μL of lysate, 1× Biomix (Bioline, London, UK) and 0.5 μM of primers (fw1 5′-GAAATCTGACATAATTTTTGCTTTC-3′; fw2 5′-AATCTTATCGTAATTCTTACTT-3′; fw3 5′-CACCTATCCTTATAATAGC-3′; fw4 5′-TGCCAGTAGGGGTGCTTC-3′; rev1 5′-TTTTTAAGTTTTGTTTAACGTTTGG-3′; Rev2 5′-AACTTATAGTTAATCCAGTACC-3′; rev3 5′-TTAATAATAAGAAATTCTAG-3′; rev4 5′-ACCCCTATTTGCTGCTGTC-3′). The following conditions were used: denaturation at 94 °C for 3 min; 30 cycles of denaturation at 94 °C for 30 s; annealing at 45 °C (fw2-rev2; fw3-rev3; fw1-rev2) at 50 °C (fw1-rev1) or 60 °C (fw4-rev4) for 30 s; and extension at 72 °C for 1 min; with the final extension step at 72 °C for 5 min. PCR products were analyzed by electrophoresis on 1.5% agarose gels.

### PFGE and southern blot analysis

PFGE and Southern Blot were performed as described in [7], to determine whether the gene was plasmid-borne or chromosomal. DNA plugs for PFGE were prepared from the *C. perfringens* strains containing the *perfrin* gene. The strains were grown in TGY and the bacterial pellets incorporated into a final agarose concentration of 1% in PFGE certified agarose (Bio-Rad Laboratories). Plugs were incubated overnight with gentle shaking at 37 °C in lysis buffer (0.5 M EDTA pH8.0, 2.5% (v/v) of 20% sarkosyl, 0.25% lysozyme, 0.2% deoxycholic acid) and subsequently incubated in 2% proteinase K buffer for 2 days at 55 °C. One third of a plug per isolate was equilibrated in 200 μL restriction buffer at room temperature for 20 min and then digested with 10 U of *Not*I at 37 °C overnight. Electrophoresis was performed in a 1% PFGE-certified gel and separated with the CHEF-III PFGE system in 0.5 × Tris-borate-EDTA buffer at 14 °C at 6 V for 20 h with a ramped pulse time of 0.5 to 17.3 s. Gels were stained in RedSafe and visualized by UV light. Mid-Range II PFG markers were used as molecular DNA ladder.

DNA probes for all Southern blot steps were labeled by PCR amplification in the presence of digoxigenin-11-dUTP (DIG; Roche Applied Science) according to the manufacturer’s recommendations. DNA probes were amplified from *C. perfringens* strain CP4 [7,14]. DNA probes for the perfrin gene were prepared with specific internal primers (fw2 5′-AATCTTATCGTAATTCTTACTT-3′; rev2 5′-AACTTATAGTTAATCCAGTACC-3′). DNA from PFGE gels was transferred to nylon membranes. DNA hybridizations and detection were performed by using the DIG labeling and CSPD substrate according to the manufacturer’s recommendations (DIG system user’s guide for filter hybridization, Roche). For Southern blot hybridizations, nylon membranes were prehybridized for at least 2 h at 42 °C in hybridization solution without labeled probe and then hybridized separately at 42 °C with specific bacteriocin DNA probe for 16 h. The membranes were washed at 68 °C under high-stringency conditions.

### Purification of recombinant perfrin

The part of the *perfrin* gene encoding the 11.5 kDa active fragment (nucleotide 301 – 621) was amplified by PCR using the following primers: fw3 5′-CACCTATCCTTATAATAGC-3′ and rev3 5′-TTAATAATAAGAAATTCTAG-3′. The PCR product was cloned into the Gateway™ entry vector pENTR/TEV/TOPO and transferred into the Gateway™ expression vector pDEST17 according to the manufacturer’s instructions (Invitrogen). The recombinant protein was purified on a nickel affinity column (His GraviTrap; GE Healthcare). This results in the 11.5 kDa active fragment with a 6xHis tag and a Tobacco Etch Virus (TEV)-cleavage site at the N-terminal part of the peptide.

### Characterization of the antimicrobial peptide

Large amounts of perfrin and rPerfrin were purified using the techniques outlined above. Thermal, pH and protease stability of the purified bacteriocin and the recombinant bacteriocin were tested. The remaining antibacterial activity after temperature, pH and protease treatment was determined by the agar spot test using dilutions of the treated (either or not recombinant) perfrin suspensions. The activity of the treated Perfrin suspensions was compared with the activity of a corresponding control (= 100%). First, thermal stability of perfrin was investigated by determination of the residual antibacterial activity after incubation at 4, 24, 37, 42, 60, 80 and 100 °C for 10, 30 and 60 min. To evaluate the influence of pH on activity, the perfrin suspension was adjusted to a pH of 2.0, 4.0, 6.0, 8.0, 10.0 and 12.0 with HCl or NaOH, mixed, incubated at room temperature for 1 h, neutralized to pH 7, and tested for activity. Effect of trypsin and proteinase K (Sigma) on perfrin activity was also tested. Each enzyme was prepared at a concentration of 10 mg/mL and added to the purified bacteriocin suspension at a final concentration of 1 and 0.1 mg/mL. After incubation for 1 h at 37 °C, the inhibitory activity was tested. The ability of the purified bacteriocin to inhibit other *C. perfringens* strains was analyzed by the agar spot test by spotting 20 μL drops on lawns of the 50 *C. perfringens* strains and compared with the antimicrobial activity of the concentrated supernatant of *C. perfringens* strain 56 and of *C. perfringens* strain 56 itself (colony).

### Killing kinetics of perfrin

To determine the effect of the bacteriocin on growing cells of a sensitive strain, an overnight culture of *C. perfringens* strain 6 was diluted in TSB and grown anaerobically at 37 °C until the absorbance reached approximately 0.1 at 600 nm. The purified bacteriocin was added and samples were taken every hour for 8 h. PBS was added as control. The colony forming units per mL (cfu/mL) were determined by anaerobic incubation of 10 fold-dilutions plated on Columbia agar plates with 5% sheep blood. The experiment was performed in triplicate.

## Results

### Purification and identification of the antimicrobial peptide produced by *Clostridium perfringens* strain 56

**Figure 1:**
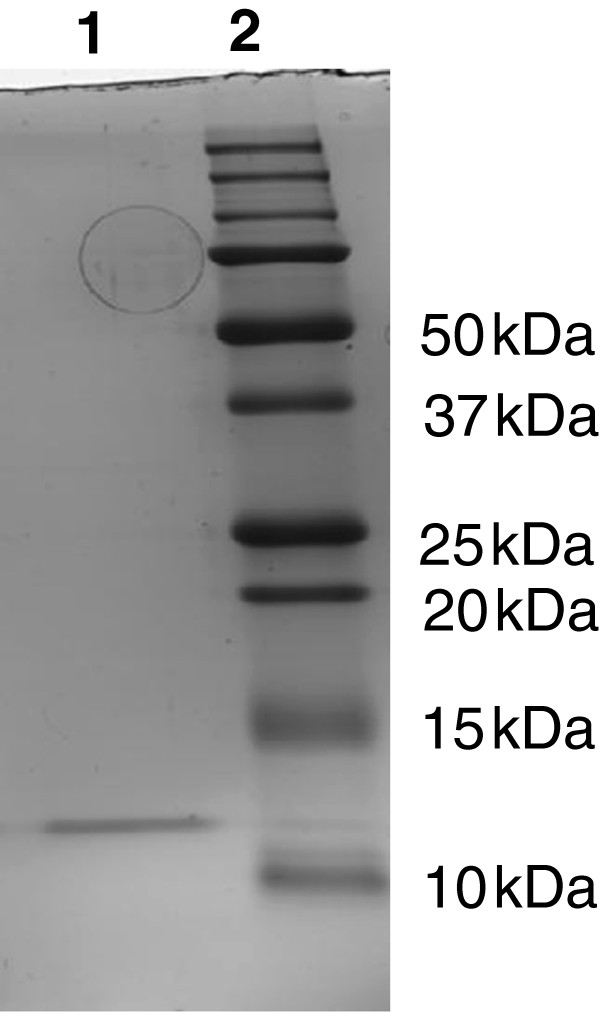
**SDS-PAGE analysis of purified perfrin from *C. perfringens *strain 56.** Lane 1: purified perfrin from *C. perfringens* strain 56; Lane 2: Precision Plus Protein Standard, All Blue.

**Figure 2:**
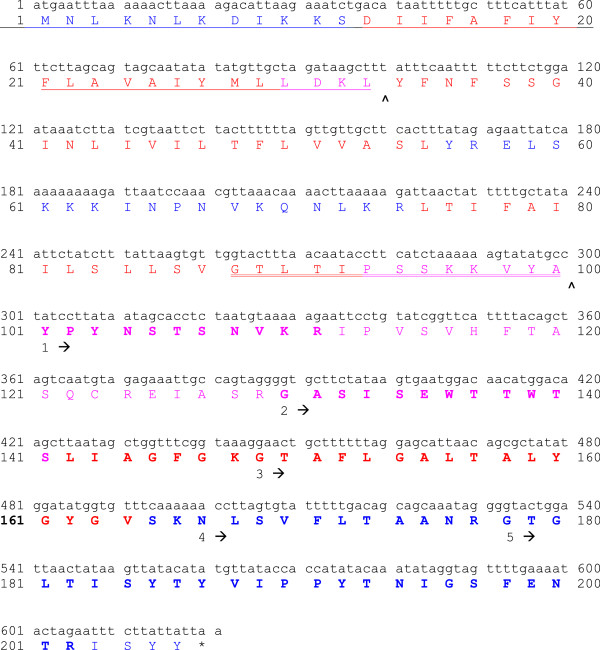
**Nucleotide and corresponding amino acid sequence of the 22.91 kDa protein.** The five amino acid sequences found by mass spectrometry are shown under the amino acid sequence (numbered from 1 to 5, starting from the arrows). The amino acids corresponding to the protein sequence are in bold type. Putative signal peptides are underlined and the putative cleavage sites are marked with an **˄**. Sequences in red, blue or pink are regions predicted by TMHMM to be located transmembrane (red), intracellular (blue) or extracellular (pink). The active bacteriocin found in the supernatant starts at amino acid position Y101.

**Figure 3:**
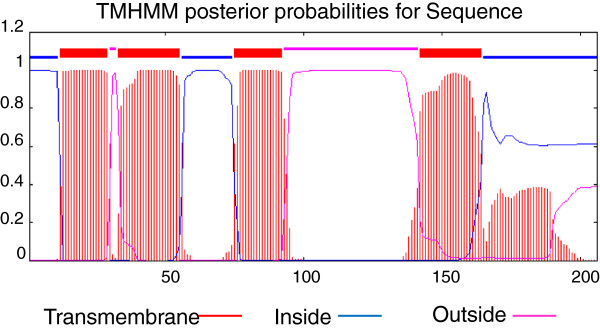
**Protein profile analysis of the 22.91 kDa protein.** The X-axis shows the amino acid position and the Y-axis indicates the probability of regions being located transmembrane (red), intracellular (blue) or extracellular (pink) predicted by TMHMM. The 11.5 kDa protein starts at the beginning of the second extracellular loop at amino acid position Y101.

Perfrin was purified by cation exchange chromatography followed by hydrophobic interaction. The fractions with antimicrobial activity showed a single band on SDS-PAGE. The molecular mass of the purified bacteriocin was approximately 12 kDa (Figure 1). The peptide mass fingerprint and MS/MS database search against the public protein databases did not allow us to identify the protein. The quality of a few MS/MS spectra of tryptic peptides was sufficient to reveal five protein fragment sequences (Figure 2). No corresponding protein sequence was found by a NCBI BLAST search. A search in the genome sequence of strain 56, using RAST, showed amino acid sequences highly similar to the five protein fragment sequences in a hypothetical protein (22.91 kDa; 207AA). All amino acid sequences found are situated in the C-terminal 106 amino acids of the protein (Figure 2). Signal-BLAST found homology in the N-terminal region with the signal sequence of *Staphylococcus aureus* enterotoxin type D, suggesting a cleavage site after amino acid at position 33 (L). Sigcleave found a signal sequence from amino acid G88 to amino acid A100 (Figure 2). The hypothetical protein is a basic protein with a pI of 9.91. Analysis of the amino acid sequence using TransMembrane prediction program TMHMM showed that the full length protein is composed of 4 transmembrane helices (Figure 3).

### Detection of *perfrin* in *C. perfringens* strains

Of the 50 poultry derived *C. perfringens* strains tested, 10 strains were positive for *perfrin*: 9 strains isolated from necrotic enteritis cases (*netB* positive) and one isolated from a healthy chicken but also positive for *netB* (Table 1). No *perfrin*-specific product was observed in any of the 45 non-poultry derived strains, which included *netB*-negative *C. perfringens* type A, B, C, D and E strains isolated from cattle, sheep, pigs, and humans.

**Table 1:** **Broiler *C. perfringens *** **strains used in the study**

	**Health**	**Toxin**		***perfrin***	**Inhibited by**			
**Strain**	**Status**	***Type***	***netB***	***(cpp)***	**Cp 56**	**SN**	**Perfrin**	**Reference**
1	Healthy	A	-	-	+	-	-	[9]
2	Healthy	A	-	-	+	+	+	[9]
3	Healthy	A	-	-	+	+	+	[9]
4	Healthy	A	-	-	+	+	+	[9]
5	Healthy	A	-	-	+	-	-	[9]
6	Healthy	A	-	-	+	+	+	[9]
7	Healthy	A	-	-	+	-	-	[9]
8	Healthy	A	-	-	+	+	+	[9]
9	Healthy	A	-	-	+	+	+	[9]
10	Healthy	A	-	-	+	-	-	[9]
11	Healthy	A	-	-	+	-	-	[9]
12	Healthy	A	-	-	+	-	-	[9]
13	Healthy	A	-	-	+	+	+	[9]
15	Healthy	A	-	-	+	+	+	[9]
16	Healthy	A	-	-	+	+	+	[9]
17	Healthy	A	-	-	+	+	+	[9]
18	Healthy	A	-	-	+	-	-	[9]
19	Healthy	A	-	-	+	-	-	[9]
20	Healthy	A	-	-	+	-	-	[9]
21	Healthy	A	-	-	+	+	+	[9]
22	Healthy	A	-	-	+	+	+	[9]
23	Healthy	A	+	+	+	-	-	[9]
24	Healthy	A	-	-	+	+	+	[9]
25	Healthy	A	-	-	+	-	-	[9]
26	Healthy	A	-	-	+	-	-	[9]
27	Healthy	A	-	-	+	+	+	[9]
28	NE	A	+	-	-	-	-	[9]
37	NE	A	+	-	-	-	-	[9]
38	NE	A	+	-	-	-	-	[9]
43	NE	A	-	-	+	+	+	[9]
48	NE	A	-	-	+	-	-	[9]
56	NE	A	+	+	-	-	-	[9]
58	NE	A	+	+	-	-	-	[9]
60	NE	A	+	-	+	+	+	[9]
61	NE	A	+	-	-	-	-	[9]
97.78247-2	NE	A	-	-	+	-	-	[8]
98.78718-2	NE	A	+	-	+	+	+	[8]
99.63206-34	NE	A	+	-	+	-	-	[8]
00.82196-2	NE	A	+	+	+	-	-	[8]
301001-1-B1	NE	A	+	+	+	-	-	[8]
200302-1-1-Ba	NE	A	+	+	+	-	-	[8]
75-659481-1	NE	A	+	+	-	-	-	[8]
70292-4	NE	A	+	+	+	-	-	[8]
75.65603-1	NE	A	+	-	+	+	+	[8]
75.65603-2	NE	A	+	-	+	-	-	[8]
75.65948-1	NE	A	+	+	-	-	-	[8]
75.65948-6	NE	A	+	+	+	-	-	[8]
97.71994-2	NE	A	+	-	+	+	+	[8]
98.73920-13	NE	A	+	-	+	+	+	[8]
00.71842-1	NE	A	+	-	+	+	+	[8]

The health status of the flock they are isolated from, the toxinotype, *netB* and *perfrin* (*cpp*) PCR results and the antimicrobial activity of *C. perfringens* strain 56 (colony), the supernatant of *C. perfringens* strain 56 (SN) and the purified perfrin are listed.

### PFGE and southern blot

The PFGE profiles of the 10 *C. perfringens* strains positive in the *perfrin* PCR revealed one to three large plasmids ranging in size from 48–73 kb. Hybridization of the *perfrin*-probe was not observed in any of the large plasmids. However, the *perfrin* probe hybridized chromosomal DNA of all the strains.

### Purification of rperfrin

The purified rperfrin has a molecular mass of approximately 16 kDa, in accordance with the molecular mass of perfrin added with the 6xHis tag and the TEV cleavage site. Purified rperfrin showed antimicrobial activity against *C. perfringens* strain 6 without cleaving the 6xHis tag.

### Characterization of the antimicrobial peptide

Perfrin and rperfrin have the same characteristics. After 30 min at 80 °C and after 10 min at 100 °C, antimicrobial activity against strain 6 was no longer present. Antimicrobial activity was most stable at neutral pH, and a decrease was detected at pH 2 and pH 12. Complete inactivation or significant reduction in antimicrobial activity was observed after treatment with trypsin and proteinase K. The antimicrobial peptide was able to inhibit the growth of 15 of the 50 *C. perfringens* strains. It did not inhibit the growth of the producer strain, strain 56, nor did it inhibit the 9 strains that were positive for the *perfrin* gene. The concentrated supernatant spotted on a lawn also inhibited 15 of the 50 *C. perfringens* strains tested but strain 56 itself (as a colony stabbed in a lawn) was able to inhibit 41 strains.

### Effect of perfrin on a sensitive strain

**Figure 4:**
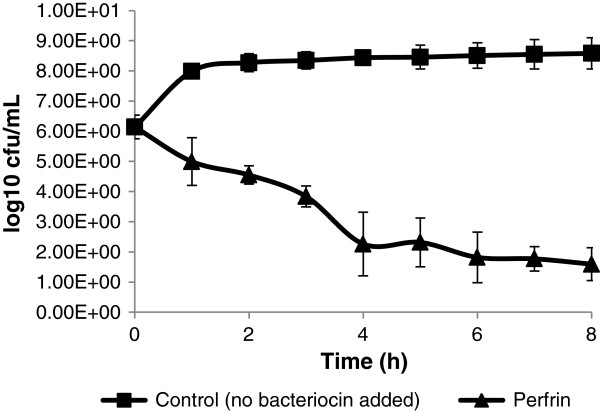
**Activity of perfrin on the viability of the sensitive *C. perfringens *strain 6.** Results shown represent the mean of three experiments, the bars represent the standard deviations. *C. perfringens* strain 6 was grown until early logarithmic phase and PBS or perfrin was added (0 h). Colony forming units were determined every hour for 8 h.

An exponentially growing culture of the sensitive *C. perfringens* strain 6 was treated with perfrin. The number of bacteria from indicator strain 6 decreased in time suggesting bactericidal activity (Figure 4).

## Discussion

The nature of the inhibitory components responsible for the intra-species growth-inhibition in necrotic enteritis associated *C. perfringens* strains was hitherto unknown. The antibacterial peptide identified in this study is a novel bacteriocin, referred to as perfrin, without homology with currently known bacteriocins or antimicrobial proteins. No sequence similarity was found with a known gene or protein in database searches. Even more, no similarity was found with gene or protein sequences of any of the 4 genome-sequenced *C. perfringens* type A strains [24-26]. The sequenced strains are two gangrene strains and a food poisoning strain isolated from humans and a strain isolated from a case of bovine clostridial abomasitis. Of the 95 *C. perfringens* strains from different origin tested, 10 were positive for the *perfrin* gene. These 10 strains were isolated from broilers and are all *netB*-positive strains. However, *netB* and *perfrin* are not located on the same genetic element since NetB is plasmid encoded [7] and perfrin not. This might be expected since a few *netB*-positive strains are *perfrin* negative (Table 1). One could expect that any strain in principle benefits from being able to inhibit closely related competitors by carrying and expressing the *perfrin* gene. The fact that perfrin is only found in *netB*-positive strains suggests that it may be important in the pathogenesis of necrotic enteritis. Recently, indications have been obtained that both plasmid and chromosomal genes contribute to the pathogenesis of necrotic enteritis [27]. The strains harboring the *perfrin* gene in combination with the *netB* plasmid probably have a selective advantage over strains only harboring the *netB* plasmid.

The newly identified bacteriocin is hypothesized to have a signal sequence in the N-terminal region (M1 – L33). This suggests that the protein is secreted. Sigcleave also shows an internal signal sequence (G88 – A100) with a cleavage site after amino acid 100 and indeed all amino acid sequences found by mass spectrometry were situated in the last 106 amino acids (Y101 – Y206) of the 22.91 kDa protein. A fragment starting at Y101 would have a molecular mass of 11.538 kDa which is in accordance with the estimated molecular mass of the purified antimicrobial peptide (Figure 1). This was confirmed by the antimicrobial activity of rperfrin which starts at Y101.

The amino acid sequence of the protein indicates the presence of four transmembrane helices (Figure 3). We can assume that the N-terminal part (three transmembrane helices) of the protein is anchored in the membrane and because of the internal signal sequence (G88 – A100), the active C-terminal part (Y101 – Y206) is released. To our knowledge, this is the first time an internal signal sequence is found in a bacteriocin.

The transmembrane helix at the C-terminal part of the 11.5 kDa part of the protein, might suggest that the antimicrobial peptide acts by pore formation. This is a feature similar to those of many characterized antimicrobial peptides from Gram-positive bacteria [13,28]. It has indeed been shown that the C-terminal region of class IIa bacteriocins forms one or two helices which penetrate the cell membrane, thereby inducing leakage and cell death [29,30]. Moreover, perfrin has bactericidal activity (Figure 4) and this is in accordance with pore formation.

*C. perfringens* strain 56 most likely produces more bacteriocins in addition to the protein identified in this work since the inhibitory spectrum of *C. perfringens* strain 56 (41/50 strains) and that of the concentrated supernatant and the purified bacteriocin (15/50 strains) are not identical. It is not unusual that a *C. perfringens* strain produces more than 1 bacteriocin simultaneously. As an example, Higa et al. [31] showed that *C. perfringens* SN-17 produced two bacteriocins in succession. Moreover, it was also shown that these bacteriocins have different inhibitory spectra. To detect the additional antimicrobial factor(s), the purification protocol needs to be adapted since these factors are not present in the concentrated supernatant prepared in the present study. There are various possibilities: maybe these bacteriocins are only secreted in co-cultures of the producing strain and a sensitive strain. This is a common feature among *Lactobacillus plantarum* bacteriocinogenic strains [32,33]. The bacteriocin might be adsorbed at the surface of the producing strain and a shift in pH could result in the release of the bacteriocin as demonstrated for different bacteriocins from lactic acid bacteria [34]. Another possibility is that the bacteriocin is secreted as inactive precursor and it needs to be processed by proteases to have antibacterial activity [35].

*C. perfringens* strain 56 and the 9 other *C. perfringens* strains that are positive for the *perfrin* gene are not sensitive to their own bacteriocin (Table 1). Genes encoding membrane-associated molecules that confer a degree of specific protection upon the producer strain have been found. For example, Diep et al. [36] showed that an immunity protein prevents *Lactococcus lactis* from being killed by its permeabilizing peptide-bacteriocin, lactococcin A, by formation of a strong complex between the receptor proteins and the bacteriocin. Moreover, it was shown that the immunity protein is only expressed if mature bacteriocin is produced or if it is present in the environment [36]. In the case of perfrin, it is tempting to speculate that protection in the producer strain may be afforded by the N-terminal fragment, which would guarantee a perfect match between bacteriocin production and protection. This, however, needs to be further investigated.

In conclusion, a novel bacteriocin called perfrin was purified from the supernatant of a necrotic enteritis inducing *C. perfringens* strain and characterized. It is the 11.5 kDa C-terminal part of a 23 kDa novel protein without any homology with currently known bacteriocins, suggesting that a new family of bacteriocins is discovered. Until now, the bacteriocin is only detected in *netB* positive *C. perfringens* strains, suggesting it might be of importance in the pathogenesis of necrotic enteritis.

## Competing interests

The authors declare that they have no competing interests.

## Authors’ contributions

LT designed the experiments, carried out most studies and drafted the manuscript. LDS participated in the purification and identification of the bacteriocin. FVN carried out the genome sequencing of *C. perfringens* strain 56. VRP carried out the PFGE and Southern Blot analysis. GVD participated in the identification of the bacteriocin. FH and RD contributed to the design of the experiments and drafting of the manuscript. JP participated in the PFGE and Southern Blot analysis. DD participated in the genome sequencing of *C. perfringens* strain 56. BD participated in the purification and identification of the bacteriocin. FVI participated in the design and coordination of the study and helped to draft the manuscript. All authors read and approved the final manuscript.
